# Increased Number of Circulating CD8/CD26 T Cells in the Blood of Duchenne Muscular Dystrophy Patients Is Associated with Augmented Binding of Adenosine Deaminase and Higher Muscular Strength Scores

**DOI:** 10.3389/fphar.2017.00914

**Published:** 2017-12-18

**Authors:** Jonathan H. Soslow, Larry W. Markham, W. Bryan Burnette, Cristi L. Galindo, Igor Feoktistov, Frank J. Raucci, Bruce M. Damon, Douglas B. Sawyer, Sergey Ryzhov

**Affiliations:** ^1^Thomas P. Graham Jr. Division of Pediatric Cardiology, Department of Pediatrics, Vanderbilt University Medical Center, Nashville, TN, United States; ^2^Division of Cardiovascular Medicine, Department of Medicine, Vanderbilt University Medical Center, Nashville, TN, United States; ^3^Division of Pediatric Neurology, Department of Pediatrics, Vanderbilt University Medical Center, Nashville, TN, United States; ^4^Departments of Radiology and Radiological Sciences, Molecular Physiology and Biophysics, and Biomedical Engineering, Vanderbilt University Medical Center, Nashville, TN, United States; ^5^Maine Medical Center, Portland, ME, United States; ^6^Maine Medical Center Research Institute, Scarborough, ME, United States

**Keywords:** Duchenne muscular dystrophy, immune response, T cells, adenosine deaminase, adenosine

## Abstract

Duchenne muscular dystrophy (DMD) is an X-linked disorder that leads to cardiac and skeletal myopathy. The complex immune activation in boys with DMD is incompletely understood. To better understand the contribution of the immune system into the progression of DMD, we performed a systematic characterization of immune cell subpopulations obtained from peripheral blood of DMD subjects and control donors. We found that the number of CD8 cells expressing CD26 (also known as adenosine deaminase complexing protein 2) was increased in DMD subjects compared to control. No differences, however, were found in the levels of circulating factors associated with pro-inflammatory activation of CD8/CD26 cells, such as tumor necrosis factor-α (TNFα), granzyme B, and interferon-γ (IFNγ). The number of CD8/CD26 cells correlated directly with quantitative muscle testing (QMT) in DMD subjects. Since CD26 mediates binding of adenosine deaminase (ADA) to the T cell surface, we tested ADA-binding capacity of CD8/CD26 cells and the activity of bound ADA. We found that mononuclear cells (MNC) obtained from DMD subjects with an increased number of CD8/CD26 T cells had a greater capacity to bind ADA. In addition, these MNC demonstrated increased hydrolytic deamination of adenosine to inosine. Altogether, our data demonstrated that (1) an increased number of circulating CD8/CD26 T cells is associated with preservation of muscle strength in DMD subjects, and (2) CD8/CD26 T cells from DMD subjects mediated degradation of adenosine by adenosine deaminase. These results support a role for T cells in slowing the decline in skeletal muscle function, and a need for further investigation into contribution of CD8/CD26 T cells in the regulation of chronic inflammation associated with DMD.

## Introduction

Duchenne muscular dystrophy (DMD) is an X-linked disorder caused by a mutation in the DMD gene, which encodes the protein dystrophin. Affecting 1 in 4,700 male births, DMD results in both cardiac and skeletal myopathy (Dooley et al., 2010). While the genetic cause of DMD is known, the underlying pathophysiology of disease is incompletely understood. Dystrophin exists along the inner surface of the plasma membrane. It is a major component of the dystrophin-glycoprotein complex (DGC), which acts as a structural link between the extracellular matrix and the cytoskeleton and also possesses cell-signaling properties (Carlson, 1998; Cohn and Campbell, 2000; Rando, 2001; Lapidos et al., 2004). The loss of dystrophin in boys with DMD disrupts the DGC and leads to destabilization of the sarcolemma, setting off a complex cascade of inflammation and immune response (Rosenberg et al., 2015). The inflammatory response, combined with calcium dysregulation, causes cycles of skeletal muscle necrosis and regeneration with eventual fibro-fatty infiltration (Carlson, 1998; Cohn and Campbell, 2000; Guiraud and Davies, 2017).

Different subpopulations of immune cells, including monocytes/macrophages, lymphocytes, eosinophils, and natural killer cells, have been found in muscles of DMD patients (Arahata and Engel, 1984; Martin et al., 2014) and dystrophin-deficient animals (Cai et al., 2000; Vetrone et al., 2009). Importantly, different subpopulations of immune cells modulate either skeletal muscle damage or repair. Thus, IFN-γ, predominantly produced from pro-inflammatory T cells, leads to suppression of M2 macrophage polarization and muscle cell proliferation (Villalta et al., 2011). In contrast, regulatory T cells (Tregs) produce IL-10 and contribute to muscle regeneration in mdx mice (Burzyn et al., 2013; Villalta et al., 2014). While immunosuppression can potentially abrogate muscle damage, it also runs the risk of suppressing immunologic repair mechanisms. Corticosteroids, which suppress inflammation and immune response, significantly delay DMD loss of skeletal muscle strength and function, but their exact mechanism remains unclear (Mendell et al., 1989; Fenichel et al., 1991; Griggs et al., 1991; Wong and Christopher, 2002; Flanigan et al., 2013; Guglieri et al., 2017). Other immunosuppressive regimens have had only limited success (Griggs et al., 1993; Kissel et al., 1993; Kirschner et al., 2010); azathioprine, for example, demonstrated a similar decrease of mononuclear infiltrates in skeletal muscle compared with corticosteroids, but no significant improvement in muscle strength (Kissel et al., 1993). The failure of some immunosuppressive medications reflects the complex role of the immune system in DMD and necessitates improved understanding of the underlying immunologic mechanisms. Identification of subpopulations of immune cells associated with the prevention of skeletal muscle damage could help in the discovery of novel, targeted therapies for prevention of DMD progression.

The goals of this project were to further explore the complex immune activation found in boys with DMD by performing systematic characterization of immune cell subpopulations obtained from peripheral blood of DMD subjects and control donors. We report for the first time that the number of CD8 cells, expressing CD26, is increased in DMD subjects compared to control donors. CD26, a cell surface glycoprotein, is known as an ADA-anchoring protein. To examine the role of CD8/CD26 T cells, we investigated the relationships between subsets of CD8 T cells and clinical parameters used to assess progression of DMD, and determined capacity of mononuclear cells to bind ADA and produce inosine in DMD subjects with increased numbers of CD8/CD26 cells.

## Methods

### Enrollment

This research protocol was approved by the Vanderbilt Institutional Review Board. The study was completed between 2012 and 2014. In accordance with the Declaration of Helsinki, participants 18 years of age and older signed informed written consent forms for the study. For those under 18 years of age, parents, or legal guardians signed informed written consents and participants signed age-appropriate informed written assent forms. DMD patients were recruited as part of a study evaluating cardiac function from the multidisciplinary Neuromuscular-Cardiology Clinic. DMD patients were over 7 years of age and the diagnosis was confirmed by either skeletal muscle biopsy or the presence of a dystrophin mutation and skeletal muscle phenotype. Healthy controls 8–18 years of age were enrolled prior to a clinically indicated treadmill test. Exclusion criteria were: (1) abnormal treadmill test, (2) presence or concern for structural or functional cardiovascular disease (congenital heart disease, cardiomyopathy, or any secondary cardiovascular disease), (3) abnormal echocardiogram, (4) arrhythmia or clinical concern for arrhythmia.

### Study procedures

Pertinent clinical data were collected from the electronic medical record for all participants with DMD. For the control participants, screening was performed prior to enrollment to assess suitability, including comprehensive questioning on past and family medical history.

### Blood collection and analysis

Venous blood (10 ml) was collected from DMD and control subjects using BD Vacutainer EDTA tubes. The total number of white blood cells (WBC) was determined after erythrocyte lysis with ammonium chloride lysing solution (150 mM NH_4_CI, 10 mM NaHCO_3_, and 1 mM EDTA, pH 7.4). Mononuclear cells (MNC) were isolated from blood on Ficoll-Paque™ Premium gradient (GE Healthcare Life Sciences, Uppsala, Sweden) within 4–6 h of drawing and cryopreserved in fetal calf serum/ dimethyl sulfoxide (9:1) using a slow temperature-lowering method (Mr. Frosty polyethylene vial holder). Cells were stored in liquid nitrogen for ≥1 week before thawing. Viability and recovery were measured using 4′,6-diamidino-2-phenylindole (DAPI) exclusion.

### Flow cytometric analysis

Flow cytometry experiments were performed as previously described (Ryzhov et al., 2017) with modifications. In brief, WBC or MNC were resuspended at the concentration of 10^6^ cell/ml and treated with Human TruStain FcX™ (Biolegend, San Diego, CA) to prevent non-specific binding followed by incubation with relevant antibodies for 25 min at 4°C. Cell-surface antigens were stained with FITC-conjugated anti-human CD3 (UCHT1), PE-conjugated CD26 (BA5b), PeCy7-conjugated CD14 (HCD14) and CD8 (HIT8a), HLA-DR-PeCy5 (L243), CD4-APC (OKT4), CD19-APC/Cy7 (HIB19), and CD16-APC/Cy7 (3G8) antibodies (all from BioLegend, San Diego, CA). Data acquisition was performed on MacsQuant Analyzer 10 (Miltenyi Biotec., Inc.).

Flow cytometry data were analyzed using WinList 5.0 software and presented in one of two ways: (1) histograms, which measure only a single parameter (e.g., expression of specific protein) or (2) dot plots, which compare two parameters simultaneously on a two-dimensional scatter plot. Axes show the number of cells (cell count) or represent fluorescence intensity (5-decade logarithmic scale) corresponded to the level of specific marker expression. Both histograms and dot plots were used to determine percentage of cells expressing specific marker using rectangular, quadrant, or freehand tools. Number of cells within specific subpopulation was calculated from total cell number and percent of corresponding cell subpopulation. Gates were set using fluorescent minus one controls or isotype-matched control IgGs. Only viable cells were analyzed. Viable and non-viable cells were distinguished using DAPI.

### Analysis of circulating TNFα, granzyme B, and IFNγ

Blood plasma was prepared and stored as previously described (Swiger et al., 2016). Plasma levels of TNFα, IFNγ, and granzyme B were measured using enzyme-linked immunosorbent assay kits (all are from Bio-techne/R&D Systems).

### Binding of adenosine deaminase

Human recombinant ADA (7048-AD-010, Bio-techne/ R&D Systems) at the final concentration of 5 μg/ml was incubated with 3 × 10^5^ mononuclear cells for 30 min in a CO_2_ incubator. Cells were washed twice with PBS/2 mM EDTA and the percentage of ADA-bound cells was determined using biotinylated anti-ADA antibody (ab34677, Abcam) in combination with Streptavidin-Phycoerythrin (Biolegend).

### Analysis of adenosine deaminase activity

After binding of ADA to the cell surface, mononuclear cells were washed twice with PBS/2 mM EDTA and resuspended in Dulbecco's Phosphate-buffered solution at the concentration of 5 × 10^4^ cells/ml. Cells were incubated in the absence or presence of 10 μM adenosine (Sigma) for 20 min in a CO_2_ incubator. The reaction was stopped by adding perchloric acid to the final concentration of 0.5 M. Supernatant was used to determine adenosine deamination to inosine using RayBio® Inosine Assay kit (Ray Biotech, Inc). Protein concentration was determined using BCA protein assay (ThermoFisher Scientific).

### Skeletal muscle strength assessment

Quantitative muscle testing (QMT) was performed by a single investigator (WBB) in participants with DMD as previously described (Posner et al., 2016). QMT is an objective, reproducible method for assessing skeletal muscle strength (Mathur et al., 2010; Lerario et al., 2012; Connolly et al., 2015). In brief, a dynamometer was used to assess total upper extremity strength, which was calculated by summing the flexion and extension values for the right and left elbows. Total lower extremity strength was calculated by summing flexion and extension values for both knees. The total QMT score was calculated as the sum of the total upper and lower extremity QMTs. All but 4 QMT measurements were performed the same day as the blood draw and none were performed longer than 3 months from the blood draw. One patient did not have QMT measured.

### Echocardiography

Echocardiograms were performed by 1 of 4 research sonographers with experience in imaging patients with DMD. Whenever possible, echocardiograms were performed supine. Post-processing included assessment of left ventricular (LV) function using fractional shortening (FS) measured from 2-dimensional images and 5/6 area length left ventricular ejection fraction (LVEF), as previously described(Lopez et al., 2010; Lang et al., 2015); these echocardiographic measures have the best accuracy and reproducibility for LV function in patients with DMD (Soslow et al., 2016). Echocardiograms were interpreted by a single investigator (JHS).

### Statistical analysis

Data were analyzed with GraphPad Prism 4.0 (GraphPad Software Inc., San Diego, CA). Normally distributed variables are expressed as mean ± SEM. Comparisons between two groups were performed using two-tailed unpaired *t*-tests. Data are expressed as median values when distributions are skewed. For variables with skewed distributions, pairwise comparisons of median values were examined using Mann–Whitney test. For continuous variables, correlation analysis was performed using a Pearson (normal distribution) or Spearman (skewed distribution correlation). A *P*-value < 0.05 was considered significant.

## Results

A total of 20 DMD participants and 10 controls were analyzed. There was no significant difference in age between DMD and controls (Table 1). As expected, controls were taller than DMD participants. There were 4 females enrolled in the control cohort; all DMD participants were male. DMD participants had decreased LVEF and FS compared with controls (49% ± 12 vs. 61% ± 5 and 26% ± 9 vs. 39% ± 4, *p* = 0.007 and *p* = 0.001, respectively). A total of 11 DMD participants (55%) had abnormal LV systolic function defined as FS < 28% or LVEF < 55%. Two DMD participants (10%) were ambulatory. The median total QMT was 55 pounds (interquartile range 39, 105) for DMD patients.

**Table 1:** Demographics and characteristics of study subjects.

	**Control (*N* = 10)**	**DMD (*N* = 20)**	***p*-value**
Age	14.4 (12.5, 16.3)	14.2 (12.0, 16.1)	0.965
Male gender	60%	100%	0.002
Height (cm)	170 (154, 174)	151 (139, 160)	0.015
Weight (kg)	65 (52, 85)	55 (40, 60)	0.131
Race			0.136
Caucasian	6 (60%)	17 (85%)	
African American	2 (20%)	2 (10%)	
Asian	0	1 (5%)	
Mixed	0	0	
Unknown	2 (20%)	0	
Hispanic	1 (10%)	3 (15%)	0.864
Ambulatory	0	2 (10%)	
Total quantitative muscle testing (QMT) (pounds)	Not tested	55 (39, 105)	
Arm QMT (pounds)	Not tested	18 (11.5, 39.5)	
Leg QMT (pounds)	Not tested	34.5 (27.5, 69)	
Current Medications:			
Corticosteroid	0	16 (80%)	
Angiotensin converting enzyme inhibitor	0	13 (65%)	
Angiotensin receptor blocker	0	5 (25%)	
Beta-blocker	0	7 (35%)	
Aldosterone inhibitor	0	0	

*Data presented as the median with interquartile range in parentheses or the total number of patients with percent of patients in parentheses. DMD, Duchenne muscular dystrophy*.

### Number of CD8 T cells is increased in DMD subjects

**Figure 1:**
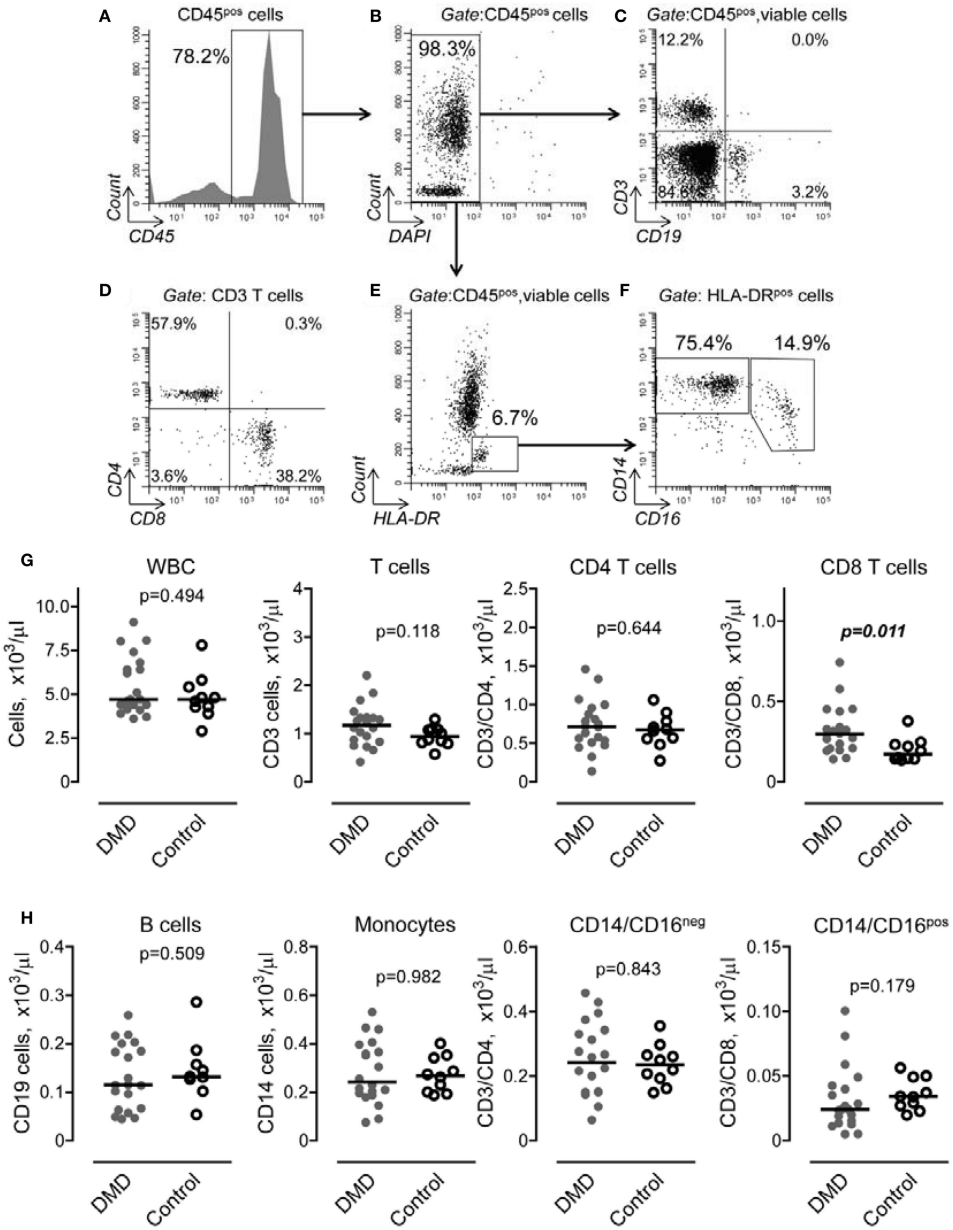
Gating strategy and flow cytometric analysis of peripheral blood lymphocytes and monocytes in Duchenne muscular dystrophy (DMD) and control subjects. **(A,B)** CD45 positive **(A)**, viable **(B)** cells were *initially* gated to *exclude non-lysed erythrocytes and dead cells from analysis*. **(C)** Subpopulation of T lymphocytes (*upper left quadrant*) and B lymphocytes (*lower right quadrant*) were distinguished by expression of CD3 and CD19 cell markers. **(D)** Subsets of CD4 (*upper left quadrant*) and CD8 (*lower right quadrant*) were determined within CD3 positive subpopulation. **(E)** Monocytes were defined as HLA-DR positive/SSA intermediate cells (*rectangular gate*). **(F)** Subpopulation of CD14^pos^CD16^neg^ and CD14^pos^CD16^pos^ monocytes were determined within HLA-DR positive/SSA intermediate cells. Representative dot plots are shown. **(G,H)** Number of cell subpopulation was calculated from total cell number and percent of corresponding cell subpopulation defined as shown in **(A–F)**. Data are presented as scatter dot plots and the horizontal line indicates the median values. Differences between DMD (*n* = *20*) and control (*n* = 0) subjects were examined using Mann–Whitney *U*-test. *P*-values are indicated.

To determine if DMD is associated with changes in systemic inflammatory response, we performed a flow cytometric analysis of lymphocyte and monocyte subpopulations in peripheral blood obtained from DMD subjects and control donors. Figure 1 illustrates our gating strategy to define subpopulations of lymphocytes and monocytes within population of CD45 positive, viable cells (Figures 1A,B). We identified phenotypically distinct cell subpopulations corresponding to CD3 T lymphocytes and CD19 B lymphocytes (Figure 1C). The subpopulation of CD3 T cells was further analyzed for CD4 and CD8 T cell subsets (Figure 1D). Subsets of CD14^pos^CD16^neg^ and CD14^pos^CD16^pos^ cells were identified within HLA-DR positive cells as shown in Figures 1E,F. Our analysis revealed that the total number of WBC was not different between the two groups (Figure 1G). However, the number of CD8 T cells was significantly increased in DMD subjects compared to controls. No differences were found in other subpopulations of WBC, including CD3/CD4, CD19 cells and subsets of monocytes (Figures 1G,H).

### Subset of CD8 cells expressing CD26, but not CD71 or CD28, is increased in DMD subjects

**Figure 2:**
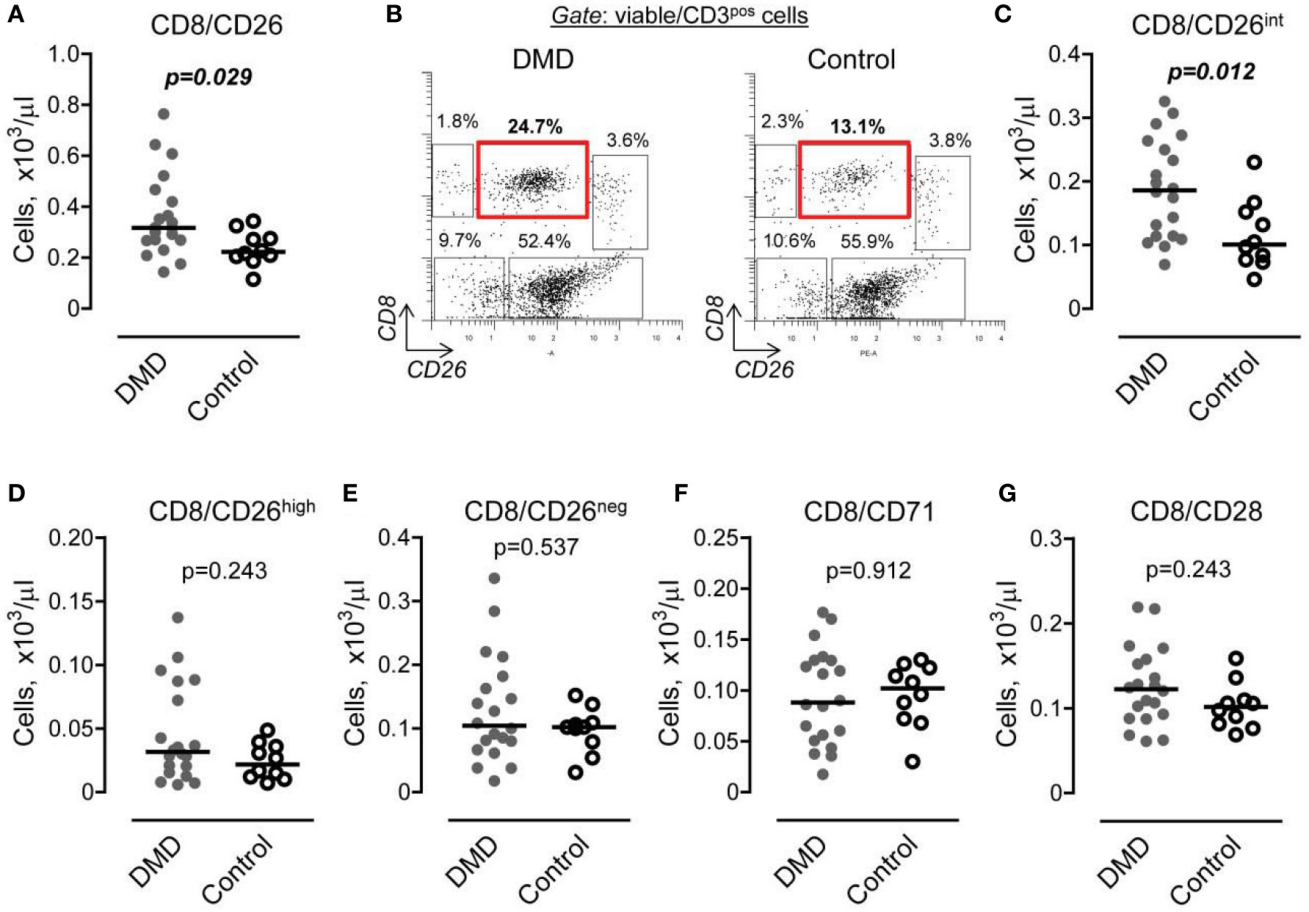
The number of CD8 cells expressing CD26 is increased in Duchenne muscular dystrophy (DMD) subjects. **(A)** Graphical representation of flow cytometry data demonstrating the number of CD8 T cells expressing CD26 in DMD (*n* = *20*) and control (*n* = *10*) subjects. *Horizontal lines indicate median values. Mann-Whitney U-test*. **(B)** Representative flow cytometric dot plots showing three subsets of CD8 cells, CD26 negative (upper left gate), CD26 intermediate (upper middle gate), and CD26 high (upper right gate) in DMD (*n* = *20*) and control (*n* = *10*) subjects. **(C–E)** Number of CD8 cells characterized by the absence or presence of CD26 expression (**C**, *intermediate subset*; **D**, *high subset*; and **E**, *negative subset*) was calculated from total number of CD3 cells and percent of corresponding subset. *Horizontal lines indicate median values. Mann-Whitney U-test*. **(F,G)** Graphical representation of flow cytometry data showing the number of CD8 cells expressing CD71 **(F)** and CD28 **(G)**. *Horizontal lines indicate medians. Mann-Whitney U-test*.

To determine whether or not changes in the number of CD8 T cells are also accompanied by T cell activation, we initially performed characterization of cell surface markers, including CD26, CD71, and CD28, which are associated with CD8 activation (Morimoto and Schlossman, 1998; Ohnuma et al., 2008). We found that the subset of CD8 positive cells expressing CD26 was increased in DMD subjects (Figure 2A). Further analysis revealed the presence of several subsets of CD8/CD26 cells, including CD26^high^, CD26^int^, and CD26^neg^ (Figure 2B). The major subset of CD8/CD26 cells was represented by CD8/CD26^int^ cells and was significantly increased in DMD vs. control subjects (Figure 2C). There were no differences in minor subsets of CD8/CD26^neg^ and CD8/CD26^high^ cells (Figures 2D,E), or in the number of CD8/CD71 and CD8/CD28 T cells (Figures 2F,G) between DMD and control subjects.

Interestingly, we found an increase in the number of CD8/CD26^int^ cells in subjects taking corticosteroids (0.21 ± 0.07 vs. 0.12 ± 0.04, *p* = 0.047). However, the uneven cohort size (*n* = 16 vs. *n* = 4, subjects taking vs. not taking corticosteroids, Table 1) necessitates further validation of this positive correlation between corticosteroids and CD8/CD26^int^ cells. There were no differences noted in subjects on or off angiotensin converting enzyme inhibitors or angiotensin receptor blockers. Subjects taking beta-blockers had a decrease in a minor subpopulation of CD8/CD26^high^ T cells (0.02 ± 0.01 vs. 0.06 ± 0.04, *p* = 0.008).

### The levels of pro-inflammatory factors secreted by CD8/CD26 cells are not increased in DMD subjects

**Figure 3:**
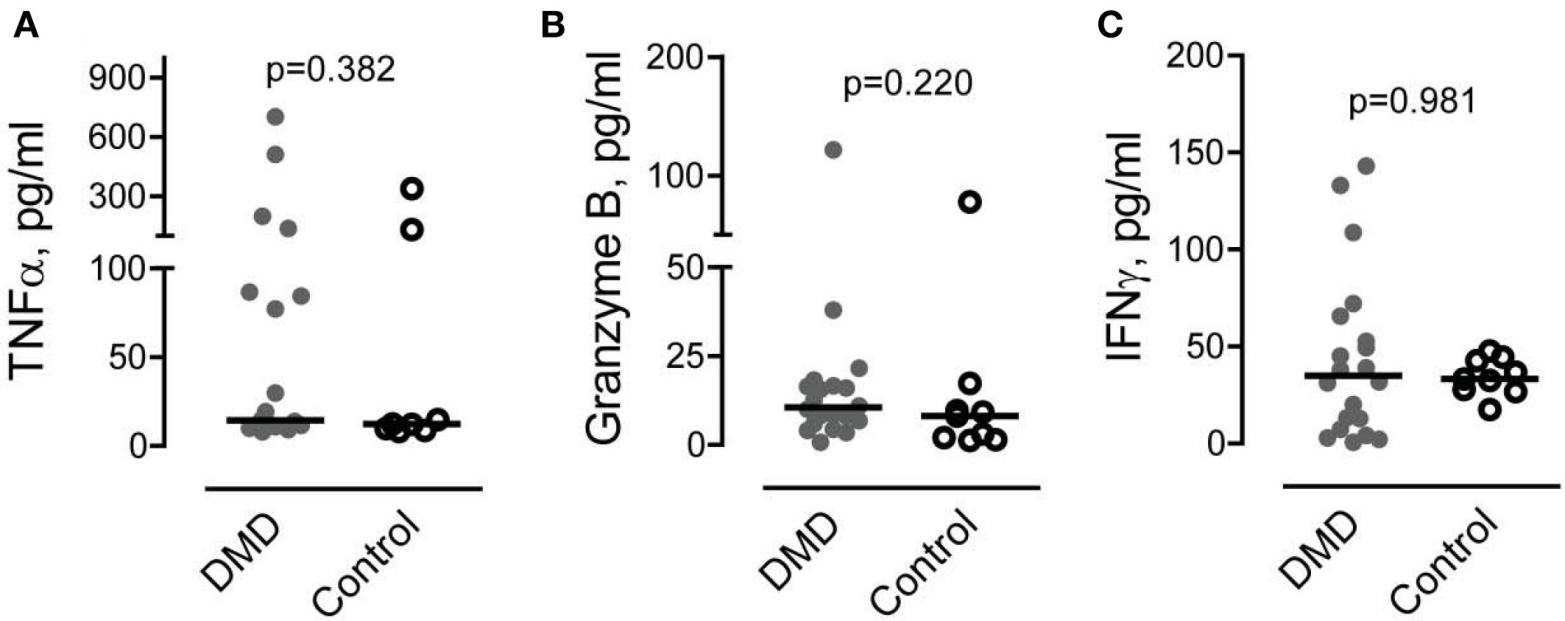
Levels of circulating cytotoxic factors associated with CD8 cell activation. **(A–C)** Tumor necrosis factor alpha (TNFα) **(A)**, granzyme B **(B)** and interferon gamma (IFNγ) **(C)** were measured in plasma obtained from Duchenne muscular dystrophy (DMD) (*n* = 20) and control (*n* = 10) subjects as described in Methods. Mann-Whitney *U* test. *P* values are indicated.

It has been recently shown that CD26 is involved in activation of CD8 T cells and stimulation of TNFα, granzyme B and IFNγ production (Hatano et al., 2013). To determine if the increased number of CD8/CD26 cells is associated with higher levels of pro-inflammatory factors, we measured TNFα, granzyme B, and IFNγ in plasma samples. As shown in Figure 3, no differences were found in the levels of pro-inflammatory factors between DMD and control subjects.

### The number of CD8/CD26 cells is associated with higher muscle strength scores

**Figure 4:**
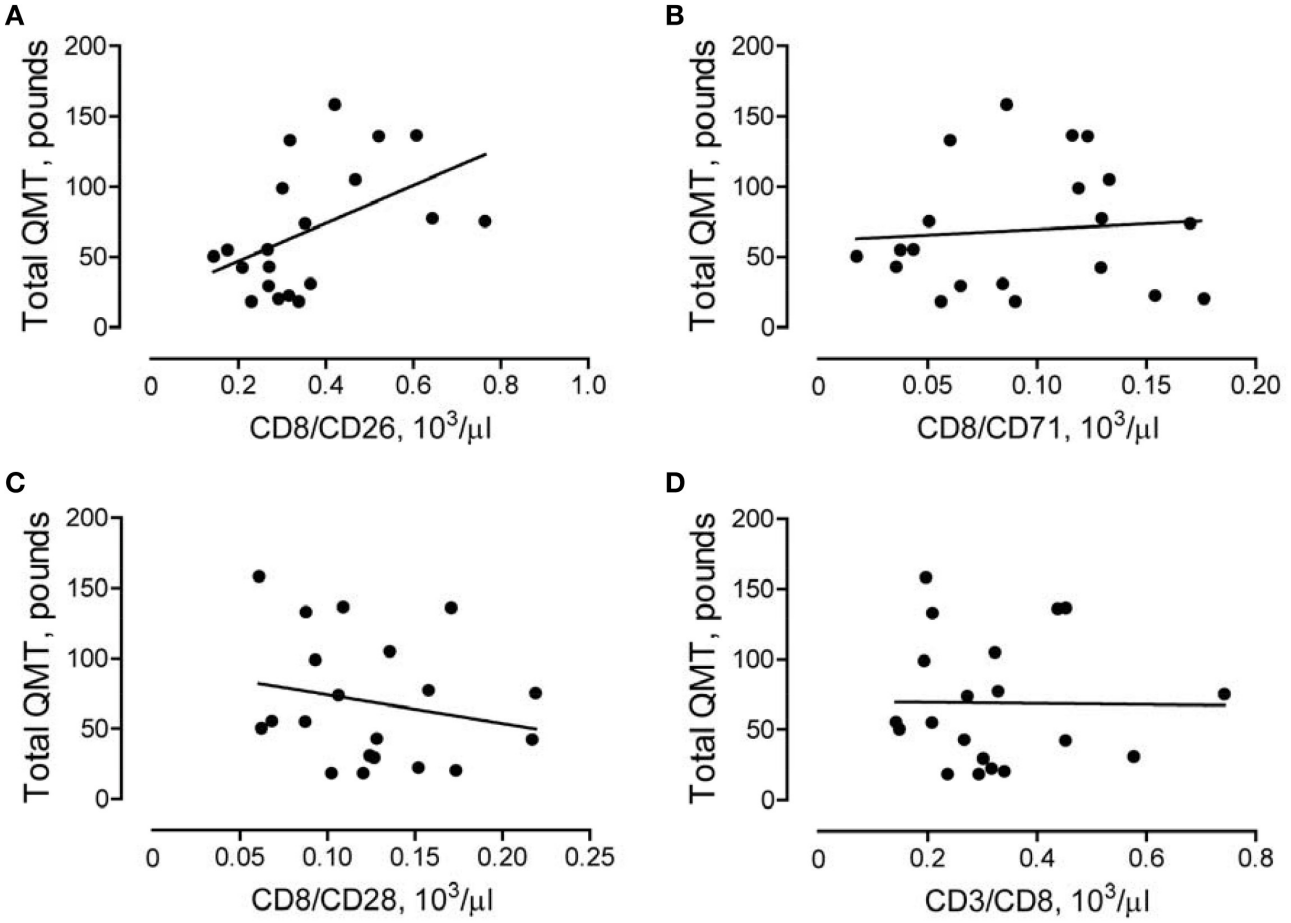
Association between number of CD8/CD26 cells and skeletal muscle strength. Quantitative muscle testing (QMT) was performed as described in Methods. The correlations between QMT and number of CD8/CD26 cells (**A**, *r*_p_ = 0.489, ***p*** = **0.028**), number of CD8/CD71 cells (**B**, *r*_p_ = 0.085, *p* = 0.719), CD8/CD28 cells (**C**, *r*_p_ = −0.211, *p* = 0.371), and CD3/CD8 cells (**D**, *r*_p_ = −0.013, *p* = 0.956) in Duchenne muscular dystrophy (DMD) subjects (*n* = *20*).

To further characterize the role of CD8/CD26 T cells in DMD subjects, we performed a correlation analysis to examine the association of these cells with clinical parameters of skeletal muscle function (QMT) and heart function (echocardiogram derived FS and LVEF). A positive correlation was found between the number of CD8/CD26 cells and the QMT score in DMD subjects (*r*_p_ = 0.489, *p* = 0.028; Figure 4A). In contrast, no significant correlations were found between CD8/CD71, CD8/CD28 subsets (Figures 4B,C), or CD3/CD8 subsets (Figure 4D), indicating the potential significance of CD26 for maintenance of muscle strength. No statistically significant correlations were identified between subsets of CD8 cells and FS or LVEF in DMD subjects (Supplementary Table 1).

### The presence of an increased number of CD8/CD26 cells is associated with higher ADA-binding capacity and deamination of adenosine to inosine

**Figure 5:**
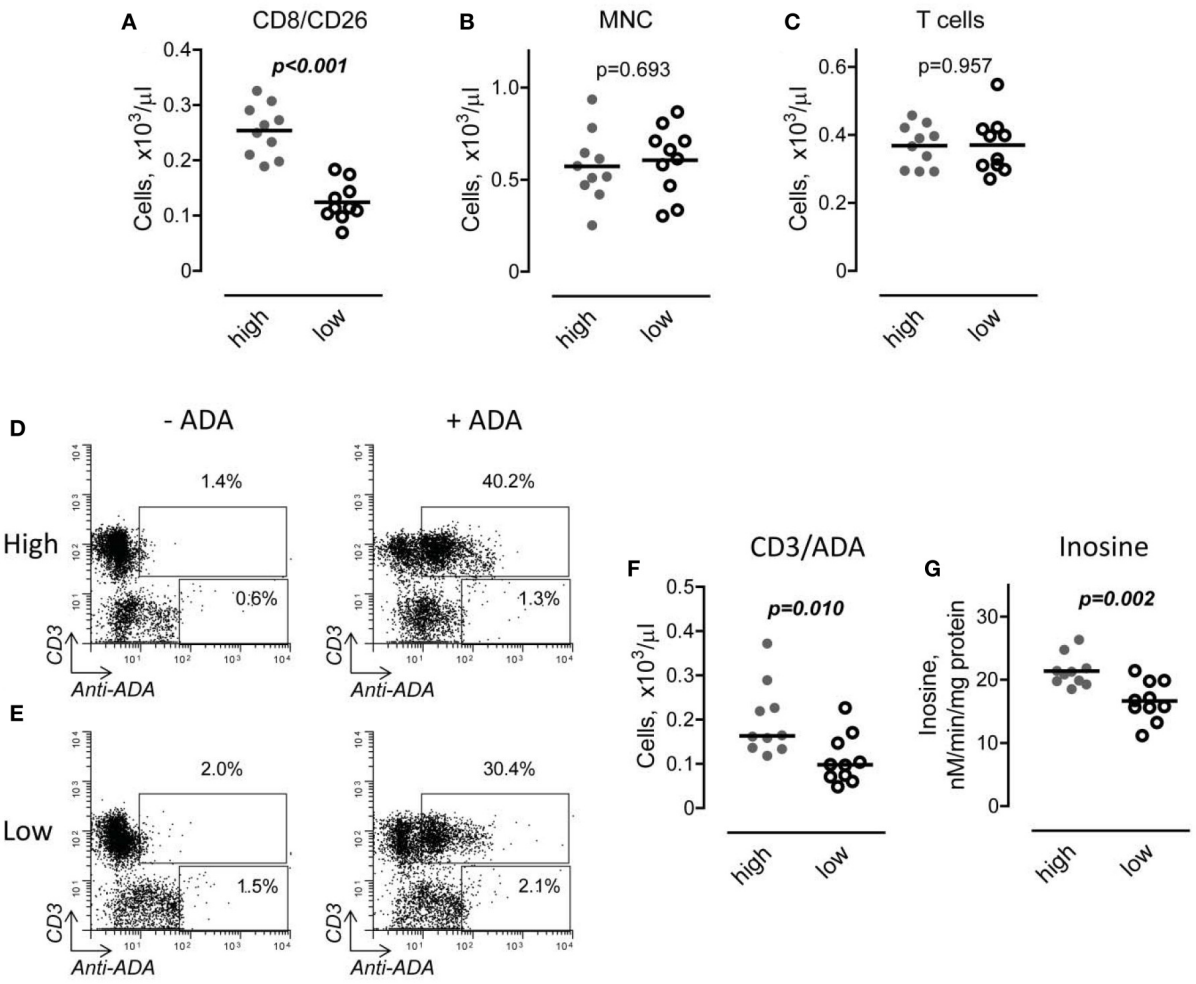
Interaction of adenosine deaminase (ADA) with CD26 in Duchenne muscular dystrophy (DMD) subjects with high and low number of CD8/CD26 cells. **(A)** DMD subjects were divided into two subgroups based on the median value of number of CD8/CD26 cells. Horizontal line indicates mean value. Unpaired *t*-test. **(B,C)** Number of mononuclear cells (MNC) **(B)** and CD3 positive T cells **(C)** in subgroups with high (*n* = *10*) and low (*n* = *10*) number of CD8/CD26 cells after isolation of MNC using Ficoll-Paque density gradient. Unpaired *t*-test. **(D,E)** Representative flow cytometric dot plots showing percent of cells which bind recombinant ADA in subgroups of subjects with high **(D)** and low **(E)** number of CD8/CD26 cells. **(F)** Graphical representation of flow cytometric data on number of cells which bind ADA. **(G)** ADA activity in MNC cell suspensions obtained from DMD subjects with high and low number of CD8/CD26 cells. Unpaired *t*-test.

In addition to being a marker of T cell activation, CD26 binds adenosine deaminase to the T cell surface. To determine if the increased number of CD8/CD26 cells is associated with a higher binding of ADA to the T cell surface, we first divided DMD subjects into two subgroups, with high and low numbers of CD8/CD26, based on the median value of number of CD8/CD26 cells as shown in Figure 5A. Then, we analyzed the capability of MNC, obtained from the peripheral blood of DMD subjects, to bind ADA. No differences were found in the total number of isolated MNC and CD3 T cells between the two subgroups (Figures 5B,C). After incubation of MNC in the absence or presence of recombinant human adenosine deaminase, the number of cells that bound ADA was determined using flow cytometry as shown in Figures 5D,E. Only CD3 T cells, but not other subpopulations of MNC, were able to bind ADA. Our analysis revealed that both percent (data not shown) and number of T cells characterized by the capability to bind ADA (ADA positive cells) was significantly higher in the subgroup of CD8/CD26^high^ compared to CD8/CD26^low^ DMD subjects (Figure 5F). To determine the functional significance of increased binding of ADA to T cells, we tested adenosine deaminase activities of MNC, incubated with ADA, in two subgroups of DMD subjects. Our data demonstrated that the level of inosine accumulation was significantly higher in the subgroup of DMD subjects with higher number of CD8/CD26 cells (Figure 5G). Thus, our data indicate that CD26 mediates binding of ADA to T cells and that the increased number of CD8/CD26 cells is associated with a higher capability of adenosine deamination to inosine in DMD subjects.

## Discussion

The immune system plays dual roles in DMD, contributing to both progression of muscle degeneration (Wehling-Henricks et al., 2008; Villalta et al., 2011) and promotion of muscle repair (Tidball et al., 2014). Animal models of muscular dystrophy reinforce this duality and demonstrate the different, sometimes diametrically opposed, effects of immune system activation (Farini et al., 2007; Villalta et al., 2009, 2014). In the current study, we investigated the association between subpopulations of immune cells and muscle strength in DMD. Our main findings are that the number of CD8 T cells expressing CD26 is increased in DMD subjects and associated with a higher muscle strength score.

DMD is characterized by intramuscular infiltration of immune cells, including T cells (Spencer and Tidball, 2001). Studies in mdx mice have shown that T cells may be involved in promotion of muscle fibrosis and eosinophilia (Cai et al., 2000; Farini et al., 2007). However, the contribution of specific subsets of CD8/CD26 cells was not determined in those studies. CD26 has been previously characterized as a co-activation marker of both CD4 and CD8 (Morimoto and Schlossman, 1998). Co-stimulation of CD8 cells with anti-CD3 and anti-CD26 resulted in the upregulation of cytotoxic factors, such as granzyme B, TNFα, and IFNγ (Hatano et al., 2013). Our studies show that the levels of these factors are similar in DMD and control subjects, suggesting no pro-inflammatory activation associated with the increased number of CD8/CD26 cells. We also found that among the three subsets of CD8/CD26 cells, only the number of CD8/CD26^int^ was significantly increased. It has been previously shown that CD8/CD26^int^ cells represent the subset of naive or central memory T cells, while both CD8/CD26^high^ and CD8/CD26^neg^ bore activated cell phenotypes (Ibegbu et al., 2009; Hatano et al., 2013). Interestingly, IL-15, which is involved in the maintenance and expansion of naïve CD8 T cells (Wallace et al., 2006), has also been shown to improve muscle strength in mdx mice (Harcourt et al., 2005).

Besides its involvement in T cells activation, CD26 serves as a receptor for ADA (Kameoka et al., 1993). There is no evidence of a direct immunomodulatory effect of ADA bound to CD26 in T cells. However, ADA may regulate immune and inflammatory responses through prevention of endogenous adenosine accumulation (Morimoto and Schlossman, 1998) and activation of purinergic receptors involved in inflammation and fibrosis (Gazzerro et al., 2015). The endogenous level of adenosine is increased in the muscles of DMD subjects (Castro-Gago et al., 1987; Camiña et al., 1995). It has been shown that adenosine can induce apoptosis in myogenic cells (Rufini et al., 1997; Ceruti et al., 2000). However, the role of adenosine in the regulation of inflammation and progression of DMD has not been well-characterized. Adenosine has been shown to promote differentiation of Th17 cells and production of proinflammatory IL-17 (Wilson et al., 2011). The intramuscular level of IL-17 mRNA is increased and correlates to muscle inflammation in subjects with DMD (De Pasquale et al., 2012). It has also been shown that early stages of DMD are associated with low intramuscular levels of ADA activity (Kar and Pearson, 1973). These findings suggest that adenosine accumulation may be an important regulator of inflammation-mediated muscle damage in DMD. We found that binding of ADA to T cells was higher in DMD subjects with an increased number of CD8/CD26 cells and associated with a higher rate of adenosine deamination to inosine and higher muscle strength score. It is possible that the positive correlation between CD8/CD26 cells and muscle strength could be explained by an increased intramuscular level of ADA bound to T cells, which is contributing to prevention of adenosine accumulation and muscle protection. Measurement of adenosine levels in blood and tissue samples from DMD subjects is necessary to directly address the involvement of adenosinergic mechanisms in effects mediated by CD8/CD26 T cells. It should be noted, however, that the determination of adenosine concentration *in vivo* is not simple. The short half-life of adenosine in peripheral blood (Möser et al., 1989) and massive release of adenosine triphosphate during sampling procedures (Macey et al., 2002) contribute to falsely elevated levels of adenosine. While these problems can be ameliorated with a freeze-clamping technique at the time of blood collection (Chen et al., 2013), this was not performed during the collection of samples in the current study.

Inosine exhibits anti-inflammatory properties, which include inhibition of cytokine and chemokine release from activated macrophages (Haskó et al., 2000; Garcia Soriano et al., 2001) and attenuation of TNFα production from LPS stimulated human neutrophils (Marton et al., 2001). Enhanced levels of inosine due to ADA-dependent deamination of adenosine to inosine in subjects with an increased number of CD8/CD26 cells may also contribute to muscle protection from invading inflammatory cells. However, the specific role of inosine in DMD has not been investigated.

The role of CD8 T cells in muscle regeneration has been recently demonstrated in a mouse model of cardiotoxin-induced injury (Zhang et al., 2014). In this model, genetic depletion of CD8 resulted in reduced chemokine ligand 2 production by T cells and impaired recruitment of macrophages into muscles. Monocytes and macrophages represent predominant subpopulations of intramuscular leukocytes (Honda et al., 1990; Brigitte et al., 2010) and can contribute to muscle protection through suppression of M1 macrophage-mediated cytotoxicity (Villalta et al., 2009). In the current study, we found that the number of CD8 T cells is increased in DMD subjects. However, no association was identified between CD8 T cells and monocytes in peripheral blood. Further study will help to determine if CD8 T cells induce intramuscular accumulation of pro-regenerative monocytes and macrophages in DMD human subjects.

In summary, our study shows that the number of CD8/CD26 T cells positively correlates to muscle strength in DMD. The binding of ADA to T cells and deamination of adenosine to inosine by MNC were significantly increased in subjects with a higher number of CD8/CD26 cells. We speculate that CD8/CD26 cells, acting through binding and delivery of ADA to skeletal muscles, may contribute to the prevention of adenosine accumulation and muscle protection. ADA-dependent regulation of adenosinergic signaling may represent a new therapeutic option to prevent loss of muscle strength and improve quality of life of patients with DMD.

## Author contributions

JS, SR, and DS: designed the study and interpreted the data; JS, DS, BD, and LM: helped organize the study, including recruitment, consent, and blood collection from subjects; SR, IF, CG, WB, LM, BD, FR, and JS: analyzed clinical and lab data; IF and CG: assisted with lab work and data analysis; JS and SR: wrote the manuscript, which was critically reviewed by DS, IF, FR, WB, BD and LM.

### Conflict of interest statement

The authors declare that the research was conducted in the absence of any commercial or financial relationships that could be construed as a potential conflict of interest.
