# Supplementary material for: Increased Number of Circulating CD8/CD26 T Cells in the Blood of Duchenne Muscular Dystrophy Patients Is Associated with Augmented Binding of Adenosine Deaminase and Higher Muscular Strength Scores
Source: Front Pharmacol. 2017 Dec 18;8:914. doi: 10.3389/fphar.2017.00914 (PMC5741593; doi:10.3389/fphar.2017.00914)
Supplement: Supplementary file 1 [file Table1.pdf]

Supplementary Table 1: Correlation of left ventricular fractional shortening (FS) and ejection fraction (LVEF) with CD8/CD26 subsets

	FS	p-value	LVEF	p-value
CD8CD26^{neg}	-0.12	0.66	0.15	0.59
CD8CD26^{int}	-0.03	0.92	0.12	0.68
CD8CD26^{high}	-0.12	0.64	0.17	0.55

Correlations performed using a Spearman's rho.
